# Supplementary material for: Practice of people towards COVID-19 infection prevention strategies in Benishangul Gumuz Region, North–West Ethiopia: Multilevel analysis
Source: PLoS One. 2022 Feb 16;17(2):e0263572. doi: 10.1371/journal.pone.0263572 (PMC8849496; doi:10.1371/journal.pone.0263572)
Supplement: S1 File — (DOCX) [file pone.0263572.s001.docx]

Supplementary – 1: List of study site (kebeles/ketenas) included in the study in Northwest Ethiopia, 2020

| **Region** | **Zone/Town administration** | **Districts/Woredas** | **Name of kebeles/Ketena** |
| --- | --- | --- | --- |
| Benishangul Gumuz | Assosa town administration | Assosa wored – 2 | Assosa town Ketena – 1 |
|  |  |  | Assosa town Ketena – 3 |
|  |  |  | Assosa town Ketena – 5 |
|  | Assosa Zone | Bambasi disrtict | Bambasi town 01 kebele |
|  |  |  | Menider – 50 kebele |
|  |  |  | Menider – 50 kebele |
|  |  | Kumruk district | Kurmuk town 01 |
|  |  |  | Belumu kebele |
|  |  |  | Dilashe kebele |
|  |  | Sherikole District | Sherikole town 01 kebele |
|  |  |  | Amorma kebele |
|  |  |  | Ashesheko kebele |
|  | Metekel Zone | Bullen district | Bullen town 02 kebele |
|  |  |  | Dobina Enkote kebele |
|  |  |  | Metina Gisa kebele |
|  |  | Wombera district | Debire Zeyite town 01 kebel |
|  |  |  | Shenikora kebele |
|  |  |  | Shanike Abiche kebele |
|  | Kemashi Zone | Kemashi district | Kemashi town 02 kebele |
|  |  |  | Checha Chugi kebele |
|  |  |  | Kobo Bedasa Kebele |
