# Supplementary material for: Practice of people towards COVID-19 infection prevention strategies in Benishangul Gumuz Region, North–West Ethiopia: Multilevel analysis
Source: PLoS One. 2022 Feb 16;17(2):e0263572. doi: 10.1371/journal.pone.0263572 (PMC8849496; doi:10.1371/journal.pone.0263572)
Supplement: S2 File — (DOCX) [file pone.0263572.s002.docx]

## Annex – 1: Research instrument for quantitative study

**Assosa University,**

**College of Health Science**

**College of Agricultural and Natural Resource**

**College of Social Science and Humanity**

**Department of interdisciplinary**

***Annex –1.1: Informed Consent***

**Research title:** Implementation and Its Effectiveness of Comprehensive Prevention Strategies of Novel Corona virus disease (COVID – 19) infection in Benishangul Gumuz Region, Northwestern Ethiopia: *Mixed method design*

**Investigators:** Mr. Muluwas Amentie, Mr. Melkamu Senbeta, Mr. Paulos Jaleta, Ass. Prof. Atnafu Morka, Dr. Nigatu Dissassa, Mr. Dula Ayana and Mr. Wagari Kelbessa

This informed consent consist of two sections: *information sheet* and *certificate of consent form*

***Section – I: Participant information Sheet***

**Introduction:**

Greeting, how are you my dear? My name is ___________ and I am working with Mr. Muluwas Amentie and his research team; who came from Assosa University, College of Health Science which is currently carrying out a research work on pandemic of coronavirus disease – 19 in Benishangul Gumuz Regional state, within the fairly scientifically sampled districts and clusters/“*kebeles”*.

**Purpose of the study**:

Coronavirus 2019 (COVID – 19) is an acute respiratory viral infectious disease in human being caused by RNA virus that belonged to the family of corona virus, transmitted by respiratory and fomites with the incubation period from 2 to 14 days. Thus, the incidence of this disease was growing exponentially and affects millions of the world population that expose thousands of peoples for death. Hence, one of the most urgent public health tasks is to prevent the spread of the virus from an epidemic source region to other regions within a country or globally using comprehensive incidence mitigation strategies. There are scarcity of evidences on the level of implementation and effectiveness of comprehensive public health intervention strategies on COVID – 19. Thus, this study will be targeted to assess the implementation and its effectiveness of comprehensive prevention strategies of Novel Corona virus disease (nCOVID – 19) in Benishangul Gumuz Regional State.

**Procedure and participation:**

The method of this research is mixed method design. All study participants eligible for the study in some villages will be selected and interviewed on current pandemic of coronavirus disease. As part of this study, we are collecting your information on socio-demographic, health related issue, knowledge, attitude and practice on prevention strategies on COVID – 19, preparedness and readiness of quarantine and treatment site, implementation of surveillance activities in the selected districts. This information enables the government and other stakeholders to develop programs to improve prevention and control strategies of COVID – 19 in the region. Be sure that your name will not be recorded and any other identifying information will be kept confidential and will not be shared with anyone else without your consent. Your participation is voluntary and you have the right not to participate fully or partially. Your decision about not to participate is respected and will not affect the health care you would normally receive.

**­­Risks and Discomfort**

There might be slight discomfort to share some personal information. However, we do not wish this to happen and you may refuse to answer any of the questions if you feel uncomfortable.

**­Benefits**

The research does not have any financially, health services and capacity building benefits. But your participation will help us to find out more about prevention strategies on COVID – 19 infections in our region and give the lesson for other regions and countries. And also gave an input for designing a strategies and formulation of infection prevention and control programs particularly for COVID – 19 based on the recommendation.

**Incentives:** We will not pay you for taking part in this study. However, we will thank you for your participation.

**Confidentiality**

The information that we collect in this study will be kept confidential. Any paper containing your name which will need to be kept for us to contact will be kept under lock and will not be given to anyone except the investigators.

**Right to refuse or withdraw**

If you do not have to take part in this research, you have a full right to with draw from the study and refuse to participant in the study. Being refusing to participate and withdraw from the study will not affect your future treatment at the health facility or elsewhere in any way. You may stop participating in the interview at any time.

**Who to contact**

The study participant has the right to ask information or question on the unclear ideas about the research context or contents before or during the research work. If you have any questions you may ask now or later, you can contact Mr. Muluwas Amentie, who was working at Assosa University, Telephone: *0913400353*. If you need further information beyond principal investigator, you can contact Assosa University.

Therefore, you may stop the interview at any time. Do you have any questions on what we talked so far? Now, do you agree to participate in the survey? Yes_____ No ______If, no respect the decision and thank his/her. If yes continue the interview.

**Name of the supervisor……………………. Signature………. Date……………..**

**Section – II: Certificate of Consent form**

**Title of research:** Implementation and Its Effectiveness of Comprehensive Prevention Strategies of Novel Corona virus disease (COVID – 19) infection in Benishangul Gumuz Region, Northwestern Ethiopia: *Mixed method design*

I, the undersigned have been well aware of this research undertaking by Assosa University which designated by principal investigator Mr. Muluwas Amentie. I have fully informed in the language I understand about the research work.

I have been informed that all the information I shall provide to the interview will be kept confidential. I understood the research work has no any risk and no compensation. I have had the opportunity to ask questions on unclear about the research before and during the research work and to contact concerned bodies. I had a right to withhold information, skip questions to answer or have been answered to on the base of my satisfaction. I consent voluntarily to be a participant in this study and understand that I have the right to withdraw from the interview at any time without in anyway affecting my right. I also agreed about the confidentiality of the responses to be at a higher possible level.

I have read this form or it has been read to me in the language I understood the condition stated above, therefore, I am will and confirm my participation by signing the consent.

Agree to participate in the study: 1. Yes 2. No. (Mark one of them for verbal consent

**Signature/Finger print of Participant**: ___________________

***For illiterate person***; Name of witness signature: _____________________ (any 3^rd^ person)

Signature: _________________.

Date: ____________________

**Signature of the interviewer:**

Name ___________________________ Signature ______________ date _______________

**Signature of Supervisors:**

Name ___________________________ Signature ______________ date _______________

**Community based questionnaire**

**Section – 1: Socio-Demographic Characteristics of Respondents**

| **S/N** | **Questions** | **Responses** |
| --- | --- | --- |
| Q_101_ | Household address | 1. Zone _________________________ 2. Woreda/sub city _________________ 3. Kebele/Ketena __________________ |
| Q_102_ | Place of residence | 1. Urban 2. Rural |
| Q_103_ | In which setting, are you currently living? | 1. Regional town 2. Zonal town 3. District /woreda town 4. Rural area/kebele town |
| Q_104_ | Gender of the respondent | 1. Male 2. Female |
| Q_105_ | What is your age? | ________________ years |
| Q_106_ | What is your religion? | 1. Orthodox 2. Muslim 3. Protestant 4. Catholic 5. Traditional belief 6. Other, Specify _____ |
| Q_107_ | What is your ethnicity? | 1. Berta 2. Amhara 3. Oromo 4. Gumuz 5. Shinasha 6. Other, specify _________ |
| Q_108_ | What is your marital status? | 1. Married 2. Single 3. Divorced 4. Widowed |
| Q_109_ | What is your educational status? | 1. Unable to read and write 2. Read and write (informal education) 3. Primary (grade 1-8) 4. High school (grade 9-12) 5. Diploma or Level I-IV 6. First Degree (BSc/BA) 7. Medical Doctor-GP 8. Medical Residents/specialize 9. Master degree and above 10. Other (specify)_____________________ |
| Q_110_ | What is your occupational status? | 1. Farmer 2. Merchant 3. House wife 4. Governmental employee 5. Private employee 6. Student 7. Other, specify______ |
| Q_111_ | Does this household own any domestic animals? | 1. Yes 2. No |
| Q_112_ | If “yes” for Q111, what type of domestic animal you own?  **(Multiple response is possible)** | 1. Cattle 2. Milk cows or bulls 3. Horses 4. Donkeys or mules 5. Goats 6. Sheep 7. Chickens 8. Dog/Cat 9. Other, specifies__________________ |

**Section – 2: Source of information on COVID – 19 infection prevention strategies**

| **S/N** | **Questions** | **Responses** |
| --- | --- | --- |
| **This section will be filled for the measurement of the sources and trust of information** | | |
| Q_201_ | Have you heard about COVID-19 disease? | 1. Yes 2. No |
| Q_202_ | Where is/are the source of information you mostly use to get information about COVID-19?  (***Multiple response is possible***) | 1. Television 2. Radio 3. Friends/peers/Neighbors 4. Health workers 5. Community and religious leaders 6. Internet-social media (Facebook, YouTube etc? 7. Others, specifies ____________________ |
| Q_203_ | Which source of information about COVID-19 do you trust?  (***Multiple response is possible***) | 1. Television 2. Radio 3. Friends/peers/Neighbors 4. Health workers 5. Community and religious leaders 6. Internet-social media (Facebook, YouTube etc? 7. Others, specifies ____________________ |

**Section – 3: Practice of people on COVID – 19 infection prevention strategies**

| **S/N** | | **Questions** | | **Responses** |
| --- | --- | --- | --- | --- |
| **This section will be filled for the measurement of self-practice on COVID – 19 prevention** | | | | |
| Q_301_ | Do you wash hands frequently with soap and water to kills the virus that causes COVID-19? | | 1. Yes 2. No | |
| Q_302_ | Do you wash hands frequently with alcohol-based hand rub kill the virus that causes COVID-19? | | 1. Yes 2. No | |
| Q_302_ | Do you maintain social/physical distance at least 2 meters that can prevent risk of infection with COVID-19? | | 1. Yes 2. No | |
| Q_303_ | Do you avoid touching eyes, nose and mouth prevent infection with COVID-19? | | 1. Yes 2. No | |
| Q_304_ | Do you cover your cough/sneezing using the bend of your elbow or a tissue prevent spread of COVID-19? | | 1. Yes 2. No | |
| Q_305_ | Do you avoid crowed places and close contact with anyone prevent risk of infection with COVID-19? | | 1. Yes 2. No | |
| Q_306_ | Do you stay at home help to prevent infections with COVID-19? | | 1. Yes 2. No | |
| Q_307_ | Do you accept/practice that isolation and treatment of people who are infected with the COVID-19 are effective ways to reduce the spread of the virus | | 1. Yes 2. No | |
| Q_308_ | Do you follow advice given by your healthcare provider can reduce the chance of acquiring COVID-19 | | 1. Yes 2. No | |
| Q_309_ | Do you follow recommended hand washing practices to prevent myself from COVID-19? | | 1. Yes 2. No | |
| Q_310_ | Do you have the resource (water, soup) to wash your hands frequently with water and soup to prevent yourself from COVID-19? | | 1. Yes 2. No | |
| Q_311_ | Do you protect yourself from crowed places and close contact with anyone to protect yourself from COVID-19? | | 1. Yes 2. No | |
| Q_312_ | Do you wear a nose mask when you go out during the COVID-19 pandemic? | | 1. Yes 2. No | |
| Q_313_ | Do you carry sanitizer during the COVID-19 pandemic? | | 1. Yes 2. No | |
| Q_314_ | Do you support if lockdown imposed in Ethiopia to control COVID-19? | | 1. Yes 2. No | |
| Q_315_ | Ethiopians greets each other by handshake followed by shoulder hit for man, cheeks touch for women, do you prefer this way of greetings in this COVID-19 pandemic? | | 1. Yes 2. No | |
| Q_316_ | Do you limit your movement during the COVID – 19 pandemic? | | 1. Yes 2. No | |
| Q_317_ | Do you have close intimation with domestic animals? | | 1. Yes 2. No | |
| Q_318_ | The way of managing domestic animals? | | 1. Intensive 2. Semi- intensive 3. Extensive | |
| Q_319_ | Do you practice hand washing before handling/feeding the animals | | 1. Yes 2. No | |
| Q_320_ | If “No” for Q320, what are your possible reasons;  **Please specify** | | _______________________________.  _______________________________. | |
| Q_321_ | Do you practice hand washing after handling/feeding the animals | | 1. Yes 2. No | |
| Q_322_ | If “No” for Q322, what are your possible reasons,  **Please specify** | | _______________________________.  _______________________________. | |
| Q_323_ | Do you use raw animal product for your household consumption? | | 1. Yes 2. No | |
| Q_324_ | If I have symptoms of COVID-19, do you isolate yourself from your family? | | 1. Yes 2. No | |
| Q_325_ | If I have symptoms of COVID-19, do you isolate yourself from your community? | | 1. Yes 2. No | |
| Q_326_ | If I have symptoms of COVID-19, do you report to quarantine site for further investigation and treatment? | | 1. Yes 2. No | |
| Q_327_ | Do you support the traffic blockage and limit the number of traveller in region to control COVID-19? | | 1. Yes 2. No | |

**Thank you very much!**

***Your participation provides valuable insights for all of us to react appropriately in the current novel coronavirus situation and to reach all citizens with useful information in a timely manner.***
