# Supplementary material for: Practice of people towards COVID-19 infection prevention strategies in Benishangul Gumuz Region, North–West Ethiopia: Multilevel analysis
Source: PLoS One. 2022 Feb 16;17(2):e0263572. doi: 10.1371/journal.pone.0263572 (PMC8849496; doi:10.1371/journal.pone.0263572)
Supplement: Attachment — Submitted filename: PONE-D-21-10875_reviewer.docx [file pone.0263572.s004.docx]

The authors have assessed the practice of community towards COVID-19 infections prevention strategies. This is a poorly designed study. This reviewer thinks the sample size considered in the present study is very low although the authors have nowhere mentioned the sample size. What were the questions in the questionnaire and what was the range of the knowledge score? The language used in the present study makes it even more difficult to understand the work done. The authors should consider a professional to improve the language and make the study understandable.
