# Supplementary material for: Practice of people towards COVID-19 infection prevention strategies in Benishangul Gumuz Region, North–West Ethiopia: Multilevel analysis
Source: PLoS One. 2022 Feb 16;17(2):e0263572. doi: 10.1371/journal.pone.0263572 (PMC8849496; doi:10.1371/journal.pone.0263572)
Supplement: Attachment — Submitted filename: Reflection to Editors comment one by one.docx [file pone.0263572.s005.docx]

**Reflection to Editor’s and Reviewers comment one by one**

General reflection on comment: Really, we are very happy and satisfied with the comment given by the reviewers and editors. Based on the comments, we have significantly changed and improved our manuscript. Then, the specific reflection on the comments were described as follow

| **S.No** | **Comments** | **Reflections** |
| --- | --- | --- |
| 1. | In your manuscript, please provide a summary table of participant demographics. | Dear my respected editor’s and reviewers, Thank you very much for your constructive comment! We included the socio-demographic table and results within the manuscript. My dear, please at *page 8* within the body of manuscript. |
| 2. | Please provide table 2 in the body of your manuscript. | Dear editor’s and reviewers, Thank you very much for your suggestion. When we revised the manuscript, formerly *Table – 2* was changed in to *Table – 3*. We included *Table – 3* in the body of manuscript according to your comment. My respected editor’s and reviewers, please see the revised and included Table – 3 at page – 12. |
| 3. | Please provide a list of the names of the 21 Kebeles that were sampled from as a supplementary file. | Dear editor’s and reviewers, Thank you very much for your guidance! According to your comment, we listed all study kebeles/ketean and attached as supplementary file. My dear, please see “*Supplementary file – 1*” |
| 4. | Thank you for stating in the text of your manuscript "ethical approval was obtained from Institutional Review Board (IRB) of Assosa University" and "written informed consent was obtained from each respondent". Please also add this information to your ethics statement in the online submission form. | Thank your dear editors and reviewers for your guidance. Hence, we included the issue of ethics in the database which found within the main document. |
| 5. | This is a poorly designed study. This reviewer thinks the sample size considered in the present study is very low although the authors have nowhere mentioned the sample size. | Dear reviewers, Thank you very much for your suggestion and guidance! We followed as your suggestion and comment, totally the manuscript was revised. Please the detail of methods in the body of manuscript *page 4 – 7*. Moreover, the sample size was calculated following scientific procedures and we used the single population proportion formula which scores the largest sample size compared with two population proportion formula which applied for associated factors. |
| 6. | What were the questions in the questionnaire and what was the range of the knowledge score? | Dear Reviewer, Thank you for your constructive comment, we revised it according to your comment, and it is shortly described in manuscript *page 11 – 12* . Around 25 questions were used to measure the level of practices and the overall practice score was generated by PCA analysis. Then, respondents who scored above or equal to the means score were rated as having good practice on COVID-19 prevention strategies. Moreover, the detail descriptions of type of questions were stated in *Table – 3*. |
| 7. | The language used in the present study makes it even more difficult to understand the work done. The authors should consider a professional to improve the language and make the study understandable. | Dear Reviewer and Editor’s, thank you very much for your constructive suggestion and comment. The manuscript is reviewed by English language expert who hold PhD in English to improve the quality of the paper and we adjusted it as per that expert’s comments. The comments were incorporated in to the manuscript |
